# First evidence of gonadal hybrid dysgenesis in Chagas disease vectors (Hemiptera, Triatominae): gonad atrophy prevents events of interspecific gene flow and introgression

**DOI:** 10.1186/s13071-023-06006-6

**Published:** 2023-10-27

**Authors:** Luísa Martins Sensato Azevedo, Natália Regina Cesaretto, Jader de Oliveira, Amanda Ravazi, Yago Visinho dos Reis, Samanta Cristina Antoniassi Fernandes Tadini, Isabella da Silva Masarin, Kelly Cristine Borsatto, Cleber Galvão, João Aristeu da Rosa, Maria Tercília Vilela de Azeredo-Oliveira, Kaio Cesar Chaboli Alevi

**Affiliations:** 1 Laboratório de Biologia Celular, Instituto de Biociências, Letras e Ciências Exatas, Universidade Estadual Paulista “Júlio de Mesquita Filho” (UNESP), Rua Cristóvão Colombo 2265, 15054-000 São José Do Rio Preto, SP Brazil; 2 Instituto de Biociências, Universidade Estadual Paulista “Júlio de Mesquita Filho” (UNESP), Rua Dr. Antônio Celso Wagner Zanin 250, Distrito de Rubião Júnior, 18618-689 Botucatu, SP Brazil; 3 Laboratório de Entomologia em Saúde Pública, Departamento de Epidemiologia, Faculdade de Saúde Pública, Universidade de São Paulo (USP), Av. Dr. Arnaldo 715, São Paulo, SP Brazil; 4 Laboratório Nacional e Internacional de Referência em Taxonomia de Triatomíneos, Instituto Oswaldo Cruz (FIOCRUZ), Av. Brasil 4365, Pavilhão Rocha Lima, Sala 505, 21040-360 Rio de Janeiro, RJ Brazil; 5 Laboratório de Parasitologia, Faculdade de Ciências Farmacêuticas, Universidade Estadual Paulista “Júlio de Mesquita Filho” (UNESP), Rodovia Araraquara-Jaú Km 1, 14801-902 Araraquara, SP Brazil

**Keywords:** Triatomines, Experimental crosses, Prezygotic barriers, Hybrid sterility

## Abstract

**Background:**

Hybridization events between *Triatoma* spp. have been observed under both natural and laboratory conditions. The ability to produce hybrids can influence different aspects of the parent species, and may even result in events of introgression, speciation and extinction. Hybrid sterility is caused by unviable gametes (due to errors in chromosomal pairing [meiosis]) or by gonadal dysgenesis (GD). All of the triatomine hybrids analyzed so far have not presented GD. We describe here for the first time GD events in triatomine hybrids and highlight these taxonomic and evolutionary implications of these events.

**Methods:**

Reciprocal experimental crosses were performed between *Triatoma longipennis* and *Triatoma mopan*. Intercrosses were also performed between the hybrids, and backcrosses were performed between the hybrids and the parent species. In addition, morphological and cytological analyzes were performed on the atrophied gonads of the hybrids.

**Results:**

Hybrids were obtained only for the crosses *T. mopan*♀ × *T. longipennis*♂. Intercrosses and backcrosses did not result in offspring. Morphological analyses of the male gonads of the hybrids confirmed that the phenomenon that resulted in sterility of the hybrid was bilateral GD (the gonads of the hybrids were completely atrophied). Cytological analyses of the testes of the hybrids also confirmed GD, with no germ cells observed (only somatic cells, which make up the peritoneal sheath).

**Conclusions:**

The observations made during this study allowed us to characterize, for the first time, GD in triatomines and demonstrated that gametogenesis does not occur in atrophied gonads. The characterization of GD in male hybrids resulting from the crossing of *T. mopan*♀ × *T. longipennis*♂ highlights the importance of evaluating both the morphology and the cytology of the gonads to confirm which event resulted in the sterility of the hybrid: GD (which results in no gamete production) or meiotic errors (which results in non-viable gametes).

**Graphical Abstract:**

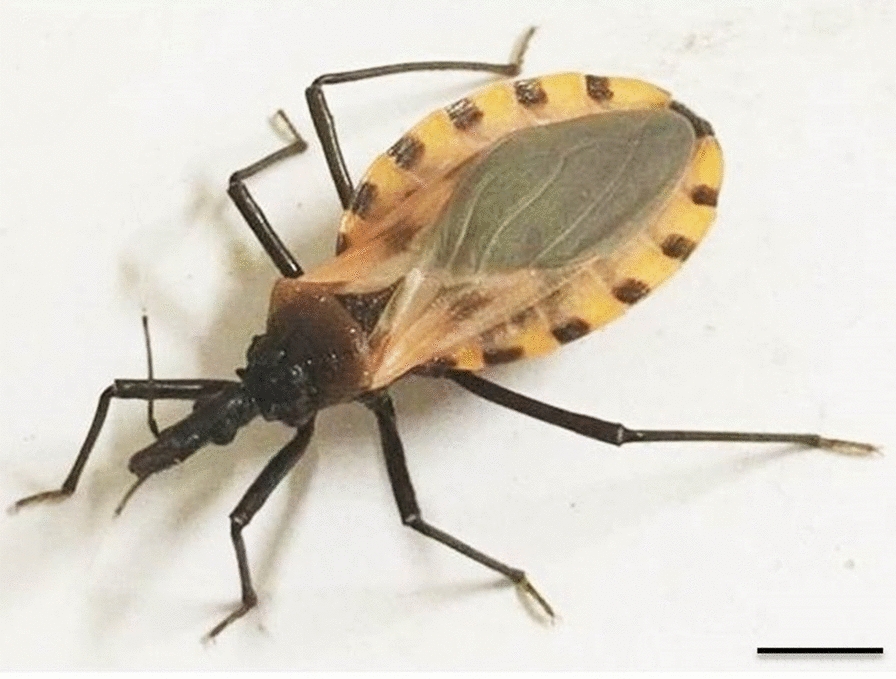

## Background

Chagas disease (CD) is a neglected disease caused by the protozoan *Trypanosoma cruzi* (Chagas, 1909) (Kinetoplastida, Trypanosomatidae) which affects about 6–7 million people worldwide [1]. Although *T. cruzi* can be transmitted in various ways, such as by blood transfusion, organ transplantation and orally [1]), the WHO considers vector transmission through the direct consumption of/contact with feces and/or urine of triatomines contaminated with *T. cruzi* to be the main transmission mode [1]. As such, vector control is considered t be the main measure to mitigate new cases of CD [1].

There are currently 160 species described in the subfamily Triatominae (157 extant species and 3 fossil species), grouped into 18 genera and five tribes (Alberproseniini, Bolboderini, Cavernicolini, Rhodniini and Triatomini) [2–6]. The Triatomini tribe is composed of nine genera (*Dipetalogaster* Usinger, 1939; *Eratyrus* Stål, 1859; *Hermanlentia* Jurberg & Galvão, 1997; *Linshcosteus* Distant, 1904; *Mepraia* Mazza, Gajardo & Jörg, 1940; *Nesotriatoma* Usinger, 1944; *Panstrongylus* Berg, 1879; *Paratriatoma* Barber, 1938; *Triatoma* Laporte, 1832) [2], with *Triatoma* being the most diversified of these and the genus with the largest number of species [2].

The genus *Triatoma* is paraphyletic [7, 8], which has led to several complexes and subcomplexes being proposed [9–11]. The Phyllosoma complex is a monophyletic grouping composed of the Phyllosoma and Dimidiata subcomplexes [9, 12]. Among the species of the Phyllosoma subcomplex, *Triatoma longipennis* (Usinger, 1939) is the main vector of *T. cruzi* in northern, western and central Mexico [13], with infection rates of between 20% and 33% [14]. To date, this species has been recorded in 11 Mexican states: Aguascalientes, Chihuahua, Colima, Durango, Guanajuato, Hidalgo, Jalisco, Michoacan, Nayarit, Sinaloa and Zacatecas [15, 16]. In contrast, among the species of the Dimidiata subcomplex, the distribution of *Triatoma mopan* Dorn et al., 2018, a species related to *Triatoma dimidiata* (Latreille, 1811) [17], is more limited than that of* T. longipennis*, with distribution restricted to the Rio Frio cave, Cayo District, Belize [17]. The authors of this latter study point out that specimens of *T. mopan* collected in the Rio Frio cave were found to be infected with *T. cruzi* [17].

Hybridization events between species of the genus *Triatoma* have been observed under natural [18–20] and laboratory conditions [21–26]. The ability to produce hybrids can influence different aspects of the parent species, and may even result in events of introgression, speciation and extinction [27]. In this context, several studies have evaluated the hybridization capacity and, above all, the reproductive barriers that prevent the formation of hybrids or result in hybrids being unviable (causing mortality, infertility or lower fitness for these organisms) [21–26, 28–30].

By studying species for the presence of interspecific barriers under laboratory conditions, it has been possible to assess the specific status of species, based on the biological concept of species [21, 25, 28–30]. Furthermore, by evaluating the ability of species to produce hybrids, the systematic and evolutionary relationship between different species can be confirmed, as hybrids, in general, are produced only among phylogenetically related species [25, 26, 28–30].

Reproductive barriers already characterized in Triatominae include the habitat [30, 31] and mechanical isolation [31, 32] as prezygotic barriers, and infeasibility [33], sterility [29, 32] and collapse [34, 35] of the hybrid as postzygotic barriers. Hybrid sterility result from unviable gametes (due chromosomal pairing [meiosis] errors) [29, 32] or the phenomenon of gonadal dysgenesis (GD) [36].

Triatomine gonads consist of two testes (in males) and two ovaries (in females) [37, 38]. The testis is an ellipsoid-shaped organ located in the abdominal region, fixed by tracheas between the second and fifth segments (almost on the side edges), located below the diaphragm (more specifically within the perivisceral sinus) [38]. It is lined with a transparent peritoneal sheath [39], which covers seven testicular follicles (sites where gametogenesis occurs) [38, 40], as well as the vessels (1 vas deferens and 7 vas efferens) and the seminal vesicle [38]. These follicles are important from a taxonomic point of view, as they vary in size between different genera [41–45].

Gonadal dysgenesis is associated with factors related to gonad atrophy in hybrids and can be unilateral or bilateral [36]. All of the triatomine hybrids analyzed so far have not presented GD [26, 29] and consequently, all recorded cases of hybrid sterility have been associated only with errors during meiosis [29, 32, 46–48]. We describe here for the first time a GD event in triatomine hybrids and highlight its taxonomic and evolutionary implications.

## Methods

### Experimental crosses

**Fig. 1:**
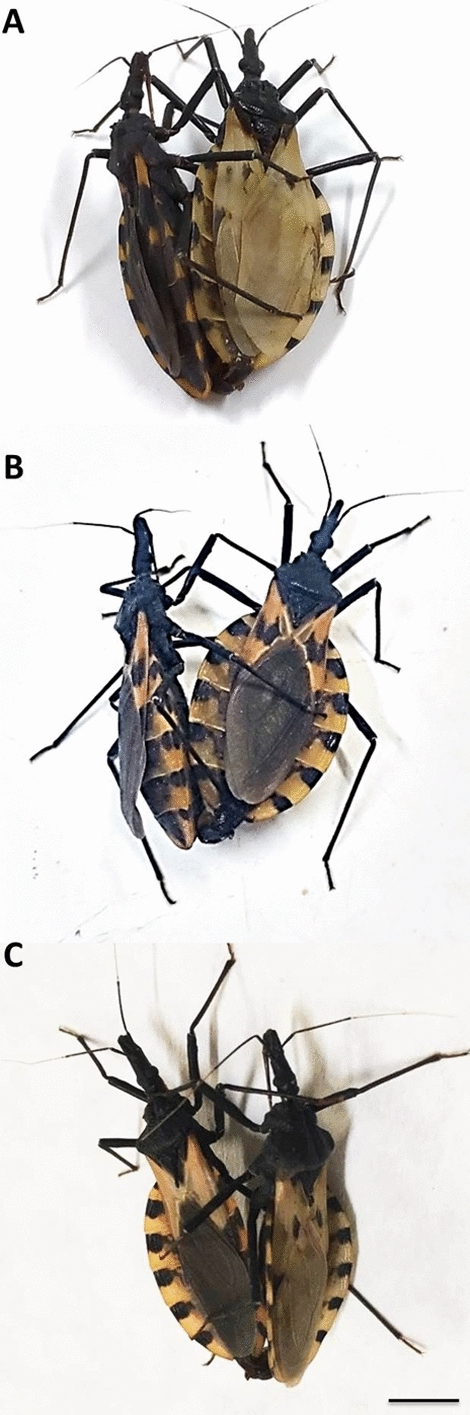
Examples of experimental crosses between *Triatoma mopan*♀ × *Triatoma longipennis*♂ (**a**), intercrossing between hybrid♀ × hybrid♂ (**b**) and backcrossing between hybrid♀ × *T. mopan*♂ (**c**). Bar: 1 cm

Reciprocal experimental crosses were performed between *T. longipennis* (origin: Mexico, Jalisco, El Grullo; colony started in March 2008) and *T. mopan* (origin: Central America, Belize, Cayo, Belmopan; colony started in August 2013) (Fig. 1a; Table 1). In addition, intercrosses were performed between the hybrids (Fig. 1b; Table 1) and backcrosses were performed between the hybrids and the parent species (Fig. 1C, Table 1). The insects used in the experiment came from colonies kept in the Triatominae insectary of the School of Pharmaceutical Sciences, São Paulo State University (UNESP), Araraquara, São Paulo, Brazil. The experimental crosses were conducted in the Triatominae insectary according to the experimental protocols of Mendonça et al. [34]. In brief, the insects were sexed as fifth instar nymphs (N5), and males and females were kept separately until they reached the adult stage to guarantee the virginity of the insects used in the crosses. For the experimental crosses, five pairs from each set were placed in plastic jars (5 cm [diameter] × 10 cm [height]) and kept at room temperature. The eggs were collected on a weekly basis and counted to evaluate the hatching rate. The eggs from the cross between *T. longipennis*♀ × *T. mopan*♂ were infertile (Table 1), and those from the cross between *T. longipennis*♂ × *T. mopan*♀ were fertile (Table 1). The N5 hybrids resulting from the cross between *T. mopan*♀ × *T. longipennis*♂ (Fig. 1a) were sexed, separated and, after the imaginal molt, five intercrosses (Fig. 1b) were performed to assess hybrid fertility (Table 1). In addition, 10 backcrosses with *T. longipennis* (5 for each direction) and 10 with *T. mopan* (5 for each direction) were also performed to assess hybrid fertility (Table 1); the eggs were collected and counted and the hatching rate evaluated in the same way as reported for the N5 cross.

**Table 1:** Experimental crosses performed between *T. mopan*, *T. longipennis* and hybrids

Crossing experiments		Number of eggs			Egg fertility,* n* (%)
		C1^a^	C2^a^	Total	
*Reciprocal crosses between parents*					
*Triatoma mopan*♀ × *Triatoma longipennis*♂		49	112	161	98 (61%)
*T. longipennis*♀ × *T. mopan*♂		61	83	144	00 (00%)
Intercrosses					
Hybrid♀^a^ × Hybrid^a^♂		00	00	00	–
*Backcrosses*					
Hybrid♀^a^ × *T. mopan*♂		00	00	00	–
*T. mopan*♀ × Hybrid♂^a^		33	35	68	00 (00%)
♀ Hybrid^b^ × *T. longipennis*♂		00	00	00	–
*T. longipennis*♀ × Hybrid♂^a^		54	59	113	00 (00%)
*Control*					
*T. mopan*♀ × *T. mopan*♂		35	42	77	58 (75%)
*T. longipennis*♀ × *T. longipennis*♂		90	87	177	138 (78%)

^a^ C1, C2 are replicates of the experimental crosses

^b^Hybrids of the cross between *T. mopan*♀ × *T. longipennis*♂

### Morphology of the gonads

Ten adult male hybrids resulting from the cross between *T. mopan*♀ × *T. longipennis*♂ were dissected at intervals of 5, 15 and 30 days after the imaginal molt. The morphology of the male gonads was analyzed under a stereomicroscope microscope (SM) (model MZ APO; Leica Microsystems GmbH, Wetzlar, Germany) fitted with the Motic Advanced 3.2 Plus Image Analysis System (Motic, Hong Kong) to evaluate the presence of GD (which may be uni- or bilateral) [36]. In addition, the gonads of 10 adult males of the parental species (*T. longipennis* and *T. mopan*) were also dissected and analyzed under the SM (control group).

### Cytological analysis

Ten male hybrids were dissected, and the testes were removed and stored in methanol:acetic acid solution (3: 1). Slides were prepared by the cell crushing technique (as described by Alevi et al. [49]), and the cytological analyses were performed with the aim to evaluate whether spermatogenesis was normal in gonads with GD, using the lacto-acetic orcein technique [49, 50]. As a control group, the gonads of 10 adult males of *T. mopan* and *T. longipennis* were also dissected and analyzed cytologically. The slides were examined by light microscopy under a Jena model Jenaval light microscope (Carl Zeiss AG, Jena, Germany) coupled to a digital camera; the Axio Vision LE 4.8 image analyzer system (Carl Zeiss AG) with a 400-fold increase was used to analyze the images.

## Results and discussion

Hybrids were obtained only for the *T. mopan*♀ × *T. longipennis*♂ crosses (Fig. 1a) (crosses between *T. mopan*♂ × *T. longipennis*♀ showed a prezygotic barrier) (Table 1). Intercrosses (Fig. 1b) were performed to evaluate the fertility of the first-generation hybrid (F1) and demonstrated that the hybrids are sterile (Table 1). To evaluate whether hybrids of both sexes were sterile, backcrosses were performed with *T. mopan* and *T. longipennis* (Table 1; Fig. 1c). None of the backcrossing directions resulted in offspring, confirming the postzygotic barrier of hybrid sterility (Table 1).

**Fig. 2:**
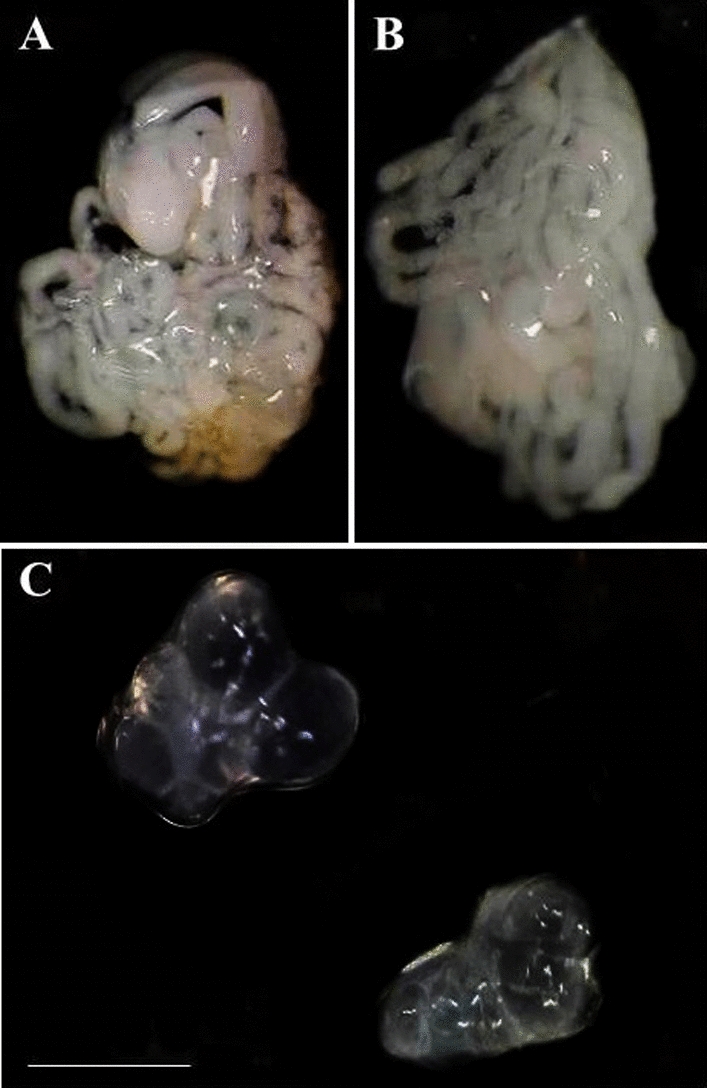
Male gonads of *Triatoma mopan* (**a**), *Triatoma longipennis* (**b**) and the hybrid (**c**). Note that the hybrid’s testes are atrophied (**c**). Bar: 10 mm

Morphological analyses of the male gonads of the hybrids (Fig. 2c) and of the parents (Fig. 2a, b) confirmed that the phenomenon which resulted in the sterility of the hybrid was bilateral GD. The gonads of the hybrids were completely atrophied (Fig. 2c), with the morphology of the testis being different morphology from that of the parents (Fig. 2a, b). The testis of the triatomine parents had seven testicular follicles (where all phases of spermatogenesis occur [51]) [38, 41–45] and a transparent peritoneal sheath [40]; in contrast, the testis of the hybrids showed only the peritoneal sheath (without seminiferous tubules) (Fig. 2c).

**Fig. 3:**
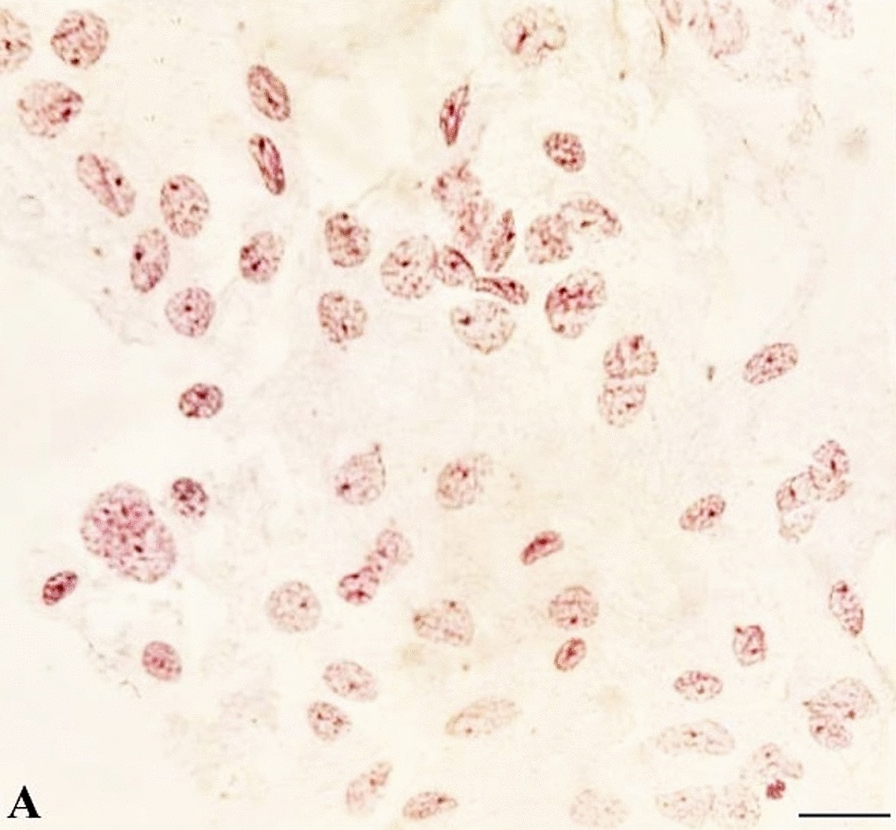
Somatic cells from the testicular peritoneal sheath of hybrids. Note the absence of germ cells. Bar: 10 μm

Cytological analyses of the testis of the hybrids confirmed GD based on the absence of germ cells and only somatic cells (with the latter forming the peritoneal sheath) (Fig. 3). In comparison, cytological analysis of the gonads of *T. mopan* and *T. longipennis* revealed the presence of spermatocytes, spermatids and spermatozoa (as has been well characterized in several studies in the subfamily Triatominae [52–55]).

In their studies on* Triatoma* spp., Perlowagora-Szumlewics and Correia [56] and Perlowagora-Szumlewics et al. [57] observed, for example, that male hybrids resulting from crossing *T. pseudomaculata* Corrêa & Espínola, 1964 × *T. sordida* (Stål, 1859), *T. pseudomaculata* × *T. infestans* (Klug, 1834), *T. pseudomaculata* × *T. brasiliensis* Neiva, 1911 and *Rhodnius prolixus* Stål, 1859 × *Rhodnius neglectus* Lent, 1954 are sterile, while females are fertile. Several interspecific crosses between *Triatoma* spp. [32], *Panstrongylus* spp. [47], *Rhodnius* spp. [46] and *Psammolestes* spp. [29] resulted in sterile hybrids. Most of these studies have cytologically analyzed the gonads of male hybrids and observed chromosomal pairing errors during meiosis, suggesting an association between the meiotic errors and hybrid sterility [29, 32, 46, 47].

Study of the interspecific reproductive barriers of insect vectors of CD has taxonomic, systematic, genetic and evolutionary value [20, 23–25, 28–30, 32–35, 46–48, 58, 59]. From a taxonomic point of view, characterization of pre- and/or postzygotic barriers confirms the specific status of the parental species [20, 23, 25, 29, 30, 32–35, 46–48, 58], based on the biological species concept [60, 61]. From a systematic point of view, in general, evolutionarily more distant species have prezygotic barriers that prevent the formation of hybrids while evolutionarily closer species can produce hybrids that will be later declined (hybrid breakdown) by postzygotic barriers [26, 30, 59]. From a genetic and evolutionary point of view, the characterization of reproductive barriers directly implies the genetic integrity of the parent species because it prevents events of interspecific gene flow and also, mainly, introgression [23, 28, 32].

The aim of crossing species belonging to two subcomplexes grouped in the Phyllosoma complex (Phyllosoma and Dimidiata subcomplexes) was to assess whether these phylogenetically related subcomplexes [7, 8, 12] are reproductively isolated or not. Thus, the production of hybrids in one direction and, subsequently, the breakdown of these hybrids by post-zygotic barriers (GD) confirm that these subcomplexes are closer in terms of a systematic perspective (as initially suggested by molecular studies [7, 8, 12]); if they had ever been distant subcomplexes, pre-zygotic barriers would be present, making hybrid formation unviable.

## Conclusions

We characterized, for the first time, GD in Triatominae and demonstrated that gametogenesis does not occur in atrophied gonads. The characterization of GD in hybrids resulting from the *T. mopan*♀ × *T. longipennis*♂ cross highlights the importance of evaluating both the morphology and the cytology of the gonads to confirm which event resulted in the sterility of the hybrid: GD (which results in no gamete production) or meiotic errors (which results in non-viable gametes).

## Data Availability

All relevant data are within the manuscript.
